# Physical activity interventions among children and adolescents in China: a scoping review through an equity lens

**DOI:** 10.1186/s12966-025-01866-w

**Published:** 2025-12-22

**Authors:** Minghui Li, Yong Liu, Yaodong Gu, Raymond Kim Wai Sum

**Affiliations:** 1 Faculty of Sports Science, Ningbo University, Ningbo, China; 2 Department of Sports and Arts, Zhejiang Wanli University, Ningbo, China; 3 Children’s Health and Exercise Research Centre, Public Health and Sports Sciences, Faculty of Health and Life Sciences, University of Exeter, Exeter, United Kingdom; 4 Department of Sports Science and Physical Education, The Chinese University of Hong Kong, Hong Kong Special Administrative Region, Shatin, Hong Kong

**Keywords:** Children, Physical activity, Health equity, Intervention

## Abstract

**Background:**

Equity in health promotion is essential to ensuring fair opportunities for children to engage in physical activity (PA). While China has implemented numerous PA interventions, little is known about how these programs address or overlook equity considerations. This scoping review synthesizes evidence on equity measures in PA interventions among Chinese children.

**Methods:**

A scoping review was conducted following the guidance of PROGRESS-Plus framework to assess equity measures across PA interventions for children in China. Published studies were analysed to identify equity-related indicators including place of residence, ethnicity, occupation, sex, education, and household income. This review was registered on OSF registries (https://osf.io/zbfpd).

**Results:**

Sixty-five individual interventions were identified. Equity considerations were rarely explicit. Most interventions were concentrated in well-developed cities, with only two conducted in rural regions. Male participants slightly outnumbered females. Children from families with lower parental education or occupational status, as well as those from ethnic minoritised groups and children who are left-behind or displaced, were consistently underrepresented. While a number of interventions focused on children with health issues, these efforts were not intentionally designed to address PA inequities.

**Conclusions:**

Equity considerations are largely absent from PA interventions for Chinese children. The disproportionate region and sex focus, combined with neglect of rural, minority, and disadvantaged groups, suggests these interventions may unintentionally widen health disparities. Future efforts should apply an equity lens in PA interventions by prioritizing underserved children and considering upstream, policy-level strategies to promote fair and inclusive PA opportunities.

**Supplementary Information:**

The online version contains supplementary material available at 10.1186/s12966-025-01866-w.

## Background

The importance of regular physical activity (PA) during childhood and adolescence are widely acknowledged [1–4]. Despite the well-documented health benefits, most children and adolescents do not engage in sufficient PA [3]. This lack of activity tends to worsen as individuals move from childhood through adolescence into adulthood [5]. Notably, PA behaviours established in youth often persist into adulthood, influencing future health conditions [6–10]. Therefore, childhood is a critical time to intervene and change behaviour before patterns become entrenched for life, potentially yielding a triple dividend – today, the future and subsequent generations [11].

However, there is growing concern that PA interventions may inadvertently exacerbate health inequities [12, 13], a phenomenon often referred to as the “inequality paradox” [14] or the “inverse care law” [15]. These “intervention-generated inequalities” can occur when interventions disproportionately benefit advantaged groups over disadvantaged ones, ultimately contributing to population-level disparities in health behaviours and outcomes [12]. Importantly, such inequalities are not inevitable. They can be mitigated when interventions are designed and implemented through an equity lens and with scale-up in mind by minimising uneven reach, ensuring high-quality implementation, and enhancing adoption and long-term sustainability [16].

China accounts for 12.9% of global adolescent population, making the promotion of PA among Chinese children essential to achieving the World Health Organization’s Global Action Plan on PA [17]. However, China faces significant equity challenges in PA participation, contributing to a widening cycle of health disparities [18]. For example, children in urban regions showed higher PA levels compared those live in suburban and rural areas [19]. A recent cross-sectional study further showed that both children who were displaced and left-behind engage in less healthy lifestyle behaviours (e.g., lower PA and higher screen time), compared with their local urban peers [20]. Socioeconomic factors also play a significant role, with household income and parental education positively related to children’s PA levels [21, 22]. Given the importance of PA for children’s physical health, these patterned differences across demographic and socioeconomic groups may exacerbate existing health inequities in China. Indeed, childhood obesity in rural and socioeconomically disadvantaged families is projected to become a major public health concern in the coming years [23].

While the Chinese Government has made extensive efforts to alleviate absolute poverty and has implemented action plans to achieve health equity by 2030 [24], it remains unclear whether PA interventions are planned and designed with an equity lens. Identifying issues related to inequities in PA interventions is a critical first step toward advancing health equity among this population in China [24–26]. Therefore, this systematic review aimed to explore: (1) To what extent is equity considered in PA interventions targeting Chinese children and adolescents? and (2) Do current PA interventions reduce or exacerbate PA disparities among this population?

### Equity measures and vulnerable groups

The PROGRESS framework provides a comprehensive and memorable structure for reporting social inequity indicators, and its measures have been meticulously selected based on evidence of their differential impact on health [27]. It includes place of residence, race/ethnicity/culture/language, occupation, gender/sex, religion, education, socioeconomic status, and social capital (collectively known as PROGRESS by acronym). To ensure a comprehensive evaluation, the present review was guided by the PROGRESS framework. We adapted these measures to better align with youth-focused research as well as the Chinese context, and the “Plus” factors were also incorporated [28]. The definition of all equity measures applied in this review are detailed in Table 1.

**Table 1:** Definition and application of equity measures

Equity measures	Definition by PROGRESS-Plus	Disadvantage groups
Place of residence	Rural, urban, and inner-city places of residence	Living in rural or less-developed cities
Ethnicity	Racial, ethnic, language, and cultural background	Minorities and displaced/left-behind children
Occupation	Refers to not only jobs, but different situations including, underemployment, informal workers, and unsafe working environments.	From families of unemployed or blue collar
Sex	NG	Females
Education	NG	From low parental education families
Household income	NG	From low-income families
Plus	Additional factors that may indicate disadvantage but are not included in the PROGRESS.	Children with health issues

PROGRESS, place of residence, race/ethnicity/culture/language, occupation, sex, religion, education, socioeconomic status, and social capital

* NG* Not given

For place of residence, inequities may raise not only between children from rural and urban areas but also among cities of varying economic development. Therefore, studies conducted in urban areas were classified according to the economic prosperity of the cities using the Chinese City Tier Classification 2025. Specifically, these studies were grouped into four categories: (1) Hong Kong, Macao or Taiwan; (2) the first-tier cities (i.e., Beijing, Shanghai, Shenzhen, and Guangzhou); (3) the new first-tier cities (e.g., Chongqing, Hangzhou, Ningbo, and Qingdao); and (4) other cities.

Ethnicity was included considering China as a multiethnic county with 56 ethnic groups, where Han majority contrasts with those minority groups. In addition, due to large-scale internal migration – where many families move from rural or less-developed cities to major coastal cities for work – approximately 90 million children are either left behind (living with grandparents) or displaced (moving to cities with their parents). These children, similar to the ethnic minority groups, represent diverse socioeconomic backgrounds, languages, and cultural and geographic contexts, and were therefore included within the ethnicity category.

As socioeconomic status is a broad term, household income was used to facilitate data charting and interpretation. Religion and social capital were excluded as they are rarely used in youth-focused studies. Moreover, we included only children living with health issues as the “Plus” measures. Accordingly, the potentially disadvantage groups including: (1) children from rural or less-developed cities; (2) ethnic minorities and children who are displaced or left-behind; (3) females; (4) children with health issues; and (5) children from disadvantaged family backgrounds (e.g., unemployment, low parental education, and low household income).

## Methods

This review aligns with the guidelines of Preferred Reporting Items for Systematic Reviews and Meta-Analyses extension for Scoping Reviews (PRISMA-SCR) [29]. The protocol of the review was registered on OSF registries (https://osf.io/zbfpd).

### Selection criteria

#### Population

To be included in this review, participants needed to be Chinese citizens with age ranged from 0 to 18 years, regardless of their health status or ethnicity.

#### Intervention

Eligible studies involved PA interventions with a duration of at least four weeks, allowing sufficient time for meaningful changes as well as consistent with a previous review [30]. We included individual level interventions that delivered PA sessions to children, either in person or online. Interventions could be multi-component, addressing PA alongside other behaviours such as nutrition or policy. Broader policy-level or environmental-only interventions were excluded.

#### Comparator

There were no restrictions on the presence or type of control groups.

#### Outcome

All types of intervention outcomes were considered.

#### Study design

Both randomised and non-randomised interventions were included. Non-intervention studies, such as cross-sectional and longitudinal studies, were excluded.

### Publication type

We only included peer-reviewed journal articles published in English. Conference papers, theses and dissertations, and review articles were not included.

### Search strategy and selection

The literature search was conducted in SPORTDiscus (EBSCOhost), Web of Science, Scopus, Medline (Ovid) and Cochrane Central Register of Controlled Trials. We searched each database from its inception (i.e., the earliest year for which indexed records are available in that database) to the date of June 2025, with no publication year limits were applied. The search strategy was developed through three key search terms: Children and adolescents, PA interventions, and China/Chinese. The full search strategy is provided in Appendix 1.

Upon removal of duplicates via Endnote (Clarivate Analytics, Philadelphia, USA), two reviewers (ML and YL) independently screened the titles and abstracts against the inclusion and exclusion criteria in a blinded manner in Rayyan [31]. Conflicts were discussed between the reviewers until consensus was reached. All studies marked as ‘Included’ or ‘Maybe’ proceeded to full-text screening, which was conducted independently and in duplicate by the same reviewers using the predefined eligibility criteria. Any discrepancies at either stage were resolved through discussion with a third reviewer (RS). Whenever additional information was needed for eligibility decisions, inquiry emails were sent to the authors. The study was excluded if no feedback was received within fourteen days. In addition to database searching, we conducted backward citation searching of all included studies and relevant reviews to identify additional eligible interventions.

### Data charting and synthesis

Study characteristics were charted using an iterative data-charting form. The charted variables included participant characteristics (age, sex, sample size, population type), study design, and key intervention parameters (type, duration, frequency, session length, modality, setting, comparator, and primary outcome). Equity-related indicators based on the PROGRESS-Plus framework (Table 1) were also charted for each study. Data charting was conducted by one reviewer (ML), and a second reviewer (YL) independently verified 20% of the charted studies to ensure accuracy and consistency. No major discrepancies were identified. Charted data were then collated descriptively and summarized using frequency counts, tables and figures, and narrative synthesis to map the distribution of equity measures across interventions.

## Results

**Fig. 1:**
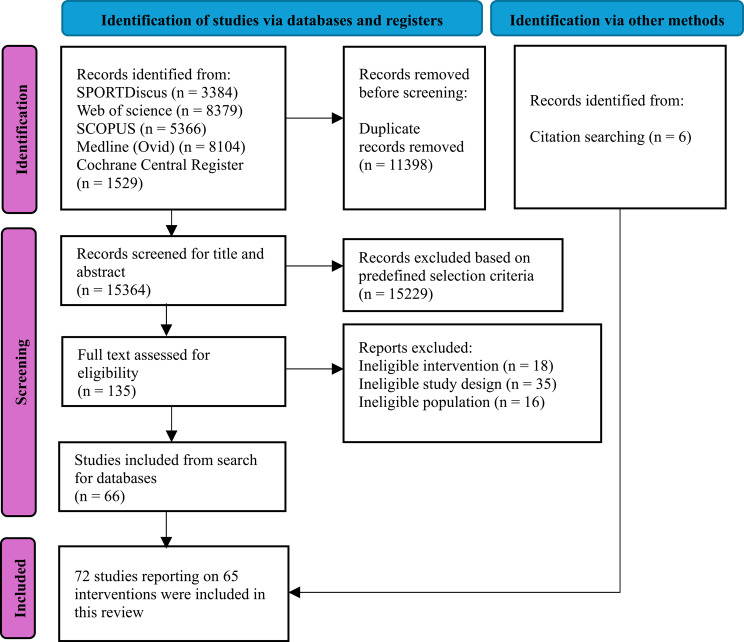
PRISMA flow diagram

The literature search and screening process is illustrated in Fig. 1. A total of 26,762 records were identified, with 15,364 records remaining for title and abstract screening after duplicate removal. We excluded 15,229 and 69 records based on predefined selection criteria. With the inclusion of additionally identified interventions, 72 studies reporting on 65 unique interventions were included in this review.

### Study characteristics

The study characteristics for the included interventions are summarised in Table 2. The majority of studies (*n* = 50, 77%) focused on children less than 13 years. Sample sizes ranged from 10 to 9585 (mean = 665, SD = 1,796), with a total of 43,230 participants. Most studies were randomised controlled trials (RCTs; *n* = 34, 52%), followed by non-randomised trials (*n* = 16, 25%), and cluster RCTs (*n* = 15, 23%). The intervention durations ranged from one month to three years, with a mean duration of 5.0 months (SD = 5.5 months). The intervention frequency varied: 18 interventions (28%) reported < 3 sessions/week, 35 (52%) ≥ 3 sessions/week, 5 (8%) reported varied frequency, and 7 (11%) did not report frequency. Session lengths ranged from 3 to 120 min (mean = 45.0 min, SD = 27.2 min; based on 46 interventions reporting data for session length). Across all studies, 35 (52%) lasted < 60 min, 18 (28%) were ≥ 60 min, and 12 (18%) did not report session duration. A variety of modalities were adopted: most studies (*n* = 42, 65%) utilised structured exercise (e.g., resistance training, ball games, dance or a combination of different exercises). By contrast, five studies (8%) employed a multi-component approach, combining PA education, training, and/or policy changes. Four studies (6%) incorporated traditional Chinese martial arts (e.g., Baduanjin and Neiyanggong) and four studies delivered their training sessions online. Most studies were delivered in schools (*n* = 45, 69%), with others conducted in home/community (*n* = 9, 14%), hospital (*n* = 6, 9%), or unspecified settings (*n* = 5, 8%). The control groups primarily received usual care or physical education (*n* = 50, 77%), while 10 (15%) received a placebo (e.g., playing card games) or light training (e.g., stretching). Three studies did not report the control condition, and one study lacked a control group. The primary outcomes varied, including health behaviours (e.g., PA engagement), physical fitness (e.g., cardiorespiratory fitness), psychological outcomes (e.g., cognitive function), as well as academic performances.

**Table 2:** Study characteristics of included studies

Author (year)	Age (SD)	Sample size (% male)	Population	Study design	Intervention length	Frequency (week)	Session length (minute)	Intervention content	Setting	Comparator	Primary outcome
Abraham et al [32]	14.3	48 (60%)	Obese	RCT	12-week	1	NG	PA education	Home	Usual care	PA
Cai et al [33]	14.0 (0.2)	222 (52%)	Healthy	RCT	12-week	3	40	Basketball, soccer, ethnic fitness exercises, and sports games	School	Usual PE	Physical fitness
Cao et al [34]	7.0 (0.4)	1854 (54%)	Healthy	Cluster-RCT	3-year	2–3	NG	Running	School&home	Usual PE	Body composition
Cao et al [35]	11.0 (0.6)	40 (50%)	Obese	RCT	12-week	3	18	Running	School	Usual PE	Body composition
Cao et al [36]	11.2 (0.7)	45 (100%)	Obese	RCT	12-week	3	11	Running	School	Usual PE	Body composition
Chan et al [37]	11.3 (3.9)	40 (90%)	Autism	RCT	4-week	2	60	Martial arts	School	Muscle relaxation	Self-control
Chang et al [38]	12.6 (0.8)	49 (73%)	Obese	RCT	12-month	6–7	15–90	Running, basketball, rope skipping, taekwondo	School	Usual PE	Insulin resistance
Chen et al [39]; Tseng et al [40];	11.3 (0.3)	52 (50%)	Healthy	RCT	8-week	5	40	Motor coordination exercises	School	Usual PE	Attention
Cui et al [41]	12.6 (0.5)	682 (52%)	Healthy	Non-RCT	4-week	1	40	Multicomponent (Nutrition, PA, SB)	School	Usual PE	PA
Dai et al [42]	16.5 (0.6)	149 (48%)	Healthy	Cluster-RCT	12-week	2	80	Ball game	School	Usual PE	Motor skills
Ding et al [43]	11.1 (0.8)	419 (54%)	Healthy	Cluster-RCT	13-week	3	7.5-8	Neuromuscular training	School	Usual PE	Injury rates
Feng et al [44]	14.0 (1.5)	181 (57%)	Postural thoracic kyphosis	RCT	8-week	2	20	Functional exercise	School	Usual PE	Postural thoracic kyphosis
Feng et al [45]	11.4 (1.7)	27 (89%)	ADHD	RCT	3-month	2–4	30–45	Balance training	Hospital	Usual care	Attention
Gu et al [46]	3–6 years	104 (48%)	Healthy	RCT	12-week	3	50	Ball game	School	Usual PE	Motor skill
Ha et al [47]	11.5 (3.1)	104 (62%)	Asthma	RCT	12-week	2	50	Aerobic, strength, balance and flexibility exercises	Hospital	Usual care	Lung function
Han et al [48]	4.2 (0.6)	110 (49%)	Healthy	Quasi	8-week	2	30	Running, climbing, jumping, throwing, balance, flexibility, and coordination	Home	Usual care	PA
Hao et al [49]	9–12 years	229 (55%)	Overweight	RCT	2-month	5	30	Rope skipping	School	Usual care	Body composition
Ho et al [50]	12.3 (0.7)	664 (41%)	Healthy	RCT	6-month	5	90	Ball game	School/community	Online health education	Well-being
Hsu et al [51]	16.5 (0.6)	54 (85%)	Mild Intellectual Disabilities	Non-RCT	12-week	3	90	Ball game	Gym	NG	Motor skill
Huang et al [52]	11.4 (0.3)	524 (51%)	Healthy	Quasi	8-week	NA	NA	Walking	Community	Waitlist control	PA
Lam et al [53]	13.0 (2.5)	70 (50%)	Cancer	RCT	24-week	1–2	60	Strength, resistance, aerobic exercises.	Home	Non-exercise games	PA
Lau et al [54]	13.1 (1.1)	252 (49%)	Healthy	Quasi	8-week	2&5	NA	Online PA and text reminder	Home	Online PA only	Programme adherence
Lau et al [55]	13.8 (0.9)	69 (52%)	Healthy	RCT	4-week	5	NA	PA homework	Home or school	Waitlist control	PA
Lau et al [56]	12.8 (0.9)	40 (0%)	Idiopathic Scoliosis	RCT	6-month	5	7	Online PA	Home	Usual care	Bone density
Li et al [57]	9.3 (0.7)	4700 (52%)	Healthy	Cluster-RCT	1-year	10	10	Multicomponent (Nutrition, PA)	School	Usual PE	Body composition
Li et al [58]	10.4	921 (53%)	Overweight/obese	Non-RCT	12-week	3	30	Multicomponent (PA, health education)	School&home	Usual PE	Body composition
Li et al [59]	6.2 (0.4)	1641 (54%)	Healthy	Cluster-RCT	12-month	NA	NA	Multicomponent (PA, health education, nutrition)	School&home	Usual PE	Body composition
Li et al [60]	14.4 (1.2)	48 (54%)	Mild to moderate mental disabilities	RCT	12-week	3	65	Running	NG	Usual care	Attention
Li et al [61]	5.5 (0.2)	87 (52%)	Healthy	RCT	10-week	2	30	Martial arts	School	Usual PE	Motor skill
Li et al [62, 63]	9.7 (0.7)	79 (41%)	Healthy	RCT	13-week	10	15		School	Usual PE	PA
Li et al [64]	8.4 (1.3)	120 (84%)	ADHD	RCT	3-month	5	30	Martial arts	Hospital	Complex	Motor skill
Liang et al [65]	8.4 (1.4)	80 (78%)	ADHD	RCT	12-week	3	60	Aerobic and neurocognitive exercise	School	Usual PE	Cognitive function
Liang et al [66]	8.4 (1.1)	30 (83%)	ADHD	RCT	12-week	3	60	Ball game	NG	Usual care	Resilience
Lin et al [67]	12.6 (0.5)	39 (41%)	Subthreshold mood syndromes	RCT	3-month	4	30	Running	School	Education	Brain structure (mood)
Liu et al [68]	6–12 years	753 (47%)	Healthy	Cluster-RCT	1-year	5	10	NG	School	Usual PE	PA
Liu et al [69, 70]	9.6 (0.4)	1392 (52%)	Healthy	Cluster-RCT	9-month	NA	NA	Multicomponent (PA, health education, PA supportive environment)	School&home	Usual PE	PA
Lu et al [71]	9.4 (1.1)	366 (52%)	Healthy	Cluster-RCT	12-week	1	NA	Multicomponent (PA, health education)	Home	Usual PE	Attitude
Luo et al [72]	12.0 (0.9)	167 (54%)	Obese	RCT	6-week	12	3	Multicomponent (PA, nutrition)	NG	Usual care	Body composition
Luo et al [73]	13.8 (0.4)	57 (51%)	Depression	RCT	12-week	4	30	Running	School	NG	Depression
Ouyang et al [74]	4–18 years	114 (71%)	Cancer	Quasi	3-month	3	30	Strength	Hospital	Usual care	PA
Shi et al [75]	5–6 years	56 (43%)	healthy	Non-RCT	12-week	3	35	Ball game	School	Usual PE	Motor skill
Tian et al [76]	12.3 (0.5)	164 (56%)	healthy	RCT	6-month	3	10	Strength	School	Light stretching	Bone health
Tseng et al [77]	10.5 (0.7)	10 (90%)	Internet addiction	Non-RCT	12-week	2	90	Ball game	School	No control	Motor skill
Wang et al [78]	10.5 (0.1)	9858 (53%)	Healthy	Cluster-RCT	1-year	NA	NA	Multicomponent (PA, PA supportive environment, health education, family events)	School&home	Usual PE	PA
Wong et al [79]	10.5 (2.1)	67 (81%)	Healthy	Non-RCT	8-week	NA	NA	Online training	Home	No control	QoL
Wang et al [80]	11.3 (0.7)	64 (56%)	Healthy	Non-RCT	16-week	NA	NA	Multicomponent (supportive environment for PA, health education, family events)	School&home	Usual PE	PA
Wang et al [81]	14.2 (0.5)	30 (73%)	Intellectual Disability	RCT	12-week	2	60	Aerobic and resistance training	School	Usual PE	Body composition
Wang et al [82]	9.2 (0.6)	2032 (49%)	Healthy	Cluster-RCT	1-year	≥ 2	120	Ball games	School	Usual PE	Academic performance
Wu et al [83]	5.5 (0.6)	239 (54%)	Healthy	Quasi	10-week	1–2	25–30	Games and aerobic exercises	School	Free play	FMS
Wu et al [84]	8.2 (1.3)	40 (75%)	Autism Spectrum Disorder	RCT	12-week	3	45	VR-Based Games	School	Usual PE	FMS
xiao et al [85]	16.7 (0.4)	40 (100%)	Tennis player	RCT	12-week	3	60	Functional training	Training club	Traditional training	Physical fitness
Xiong et al [86]	4.7	39 (49%)	Healthy	Quasi	3-month	5	30	Tug games, locomotion activities, and soccer	Day care centre	Free play	Executive Function
Xu et al [87–89]; wang et al [90]	7–13 years	9867 (48%)	Healthy	Cluster-RCT	1-year	10	10	Multicomponent (PA, PA supportive environment, health education, family events)	School	Usual PE	Body composition
Yan et al [91]	12.1	664 (53%)	Healthy	Cluster-RCT	8-week	2	40	PE sessions by trained mentor	School	NA	Enjoyment
Yu et al [92]	9.8 (0.7)	171 (80%)	Obese	Cluster-RCT	8-month	5	20–40	Strength training, rope skipping, badminton, running	School	Usual PE	Cardiovascular health
Zhang et al [93]	15.3 (0.5)	460 (43%)	Healthy	Quasi	1-year	2	90	Ball game	School	Usual PE	Academic achievement
Zhang et al [94]	12.1 (0.3)	51 (47%)	Healthy	RCT	8-week	1	45	Multicomponent (Ball game, health education)	School	Usual PE	Psychological constructs
Zhang et al [95]	8.5 (1.5)	43 (79%)	ADHD	RCT	12-week	3	60	Aerobic, resistance	Hospital	Usual PE	QoL
Zhang et al [96]	7.8 (0.7)	357 (50%)	Healthy	Quasi	10-week	1–5	10–30	Strength training, martial arts, games, PL-related education	School	Usual PE	Physical fitness
Zhang et al [97]	5–6 years	63 (48%)	Healthy	RCT	12-week	3	60	Recreational gymnastics activities	School	Educational cartoons	Executive Function
Zhao et al [98]	13.5 (0.6)	141 (100%)	Healthy	RCT	10-week	3	20–25	Strength training	School	Usual PE	Physical fitness
Zhao et al [99]	10.8 (1.1)	42 (100%)	Intellectual Disabilities	RCT	12-week	3	60	Aquatic	Disability training centre	Free play	Balance and strength
Zhou et al [100]	12.7 (0.6)	680 (53%)	Healthy	Cluster-RCT	8-month	1	45	Multicomponent (quality PE, PA supportive environment, health education)	School	Usual PE	Physical fitness
Zhou et al [101]	10.5 (1.4)	305 (52%)	Healthy	Non-RCT	1.5-year	2	90	Dance	School	No control	Academic self-efficacy
Zhou et al [102]	10.2 (0.5)	1125 (56%)	Healthy	Cluster-RCT	1-year	NA	NA	Multicomponent (supportive environment for PA, health education, fun events, family activities)	School&home	Usual PE	Obesity prevalence

*SD* Standard deviation, *RCT* Randomised controlled trial, *PE* Physical education, *NG* Not given, *ADHD* Attention deficit hyperactivity disorder, *PA* Physical activity, *Quasi* Quasiexperimental design, *VR* Virtual reality, *QoL* Quality of life, *FMS* Fundamental movement skill

### Equity measures

**Fig. 2:**
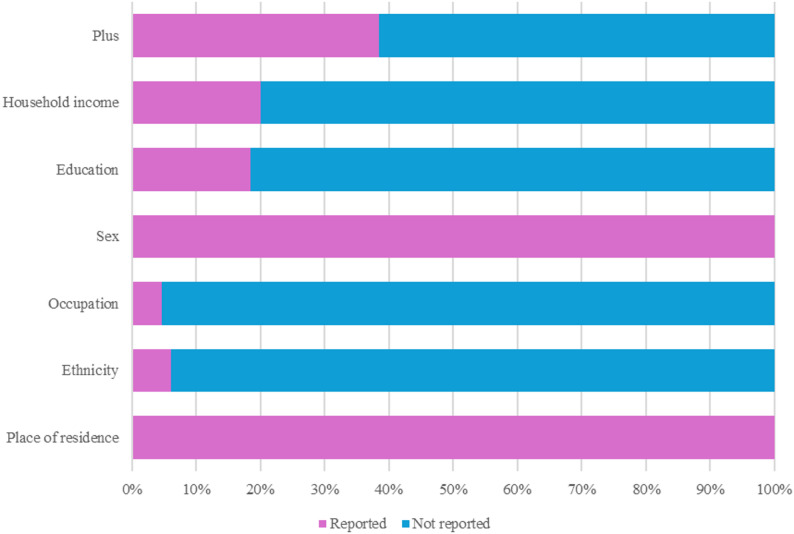
Summary of equity measures for included studies

An overview of equity measures reported in the included studies is displayed in Fig. 2. Overall, no study was conducted with a purpose or consideration of PA equity. Regarding PROGRESS-Plus measures, all studies reported data related to place of residence and sex (*n* = 65, 100%). Ethnicity (*n* = 4, 6%), occupation (*n* = 3, 5%), education (*n* = 12, 18%) and household income (*n* = 13, 20%) were rarely considered across the studies. By contrast, information on participants with health issues was more commonly reported (*n* = 25, 38%), such as children living with overweight and obesity or those with specific diseases or disorders.

### Place of residence

**Fig. 3:**
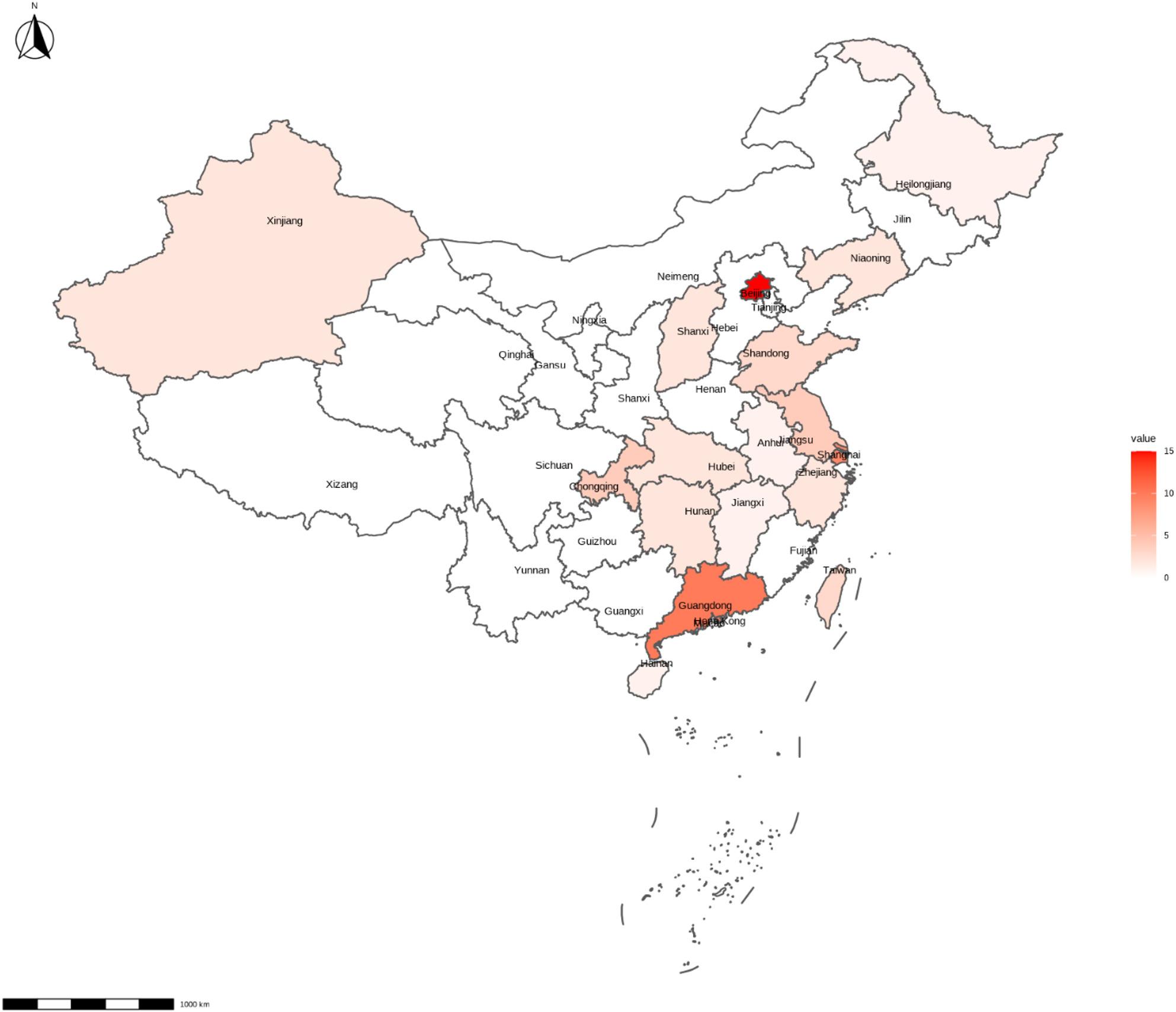
The distribution of the location of included studies

Figure 3 illustrates the distribution of the location of included studies. Overall, most studies were conducted in eastern China, primarily in highly developed cities or provinces. Only two studies took place in rural area [49, 101]. Similarly, the majority of studies were implemented in well-developed cities/regions. Thirteen studies (20%) were conducted in Hong Kong, Macao or Taiwan, almost half of the studies (*n* = 32, 49%) were delivered in First Tire cities and nine studies (14%) were carried out in the New First Tire cities. There are only eleven studies (17%) were conducted in second or lower tier cities.

### Ethnicity

There are only three studies (6%) provided the ethnicity of participants. Two (3%) were conducted among Han population [38, 94], and one (2%) among the Uyghur population [33]. Additionally, one (2%) study included children who only speak Cantonese and read Chinese [53].

### Occupation

Only three studies (5%) reported the occupation status of participants’ parents. Specifically, one study (2%) categorised parents’ occupation into three levels – executives, staff members, service workers – with most parents falling into the first two levels (*n* = 251, 172, and 63, respectively) [52]. The other two studies (3%) [79, 80] reported whether the parents were employed, showing a small number of unemployment (*n* = 29, 15%).

### Sex

All studies specified participants’ sex (i.e., male or female). Overall, males (*n* = 22398, 52%) slightly outnumbered females. Three studies (5%) included exclusively male participants [36, 98, 99], while one study (2%) included only females [56].

### Education

Parents’ education level was considered in twelve studies (18%). Among them, two studies (3%) indicated that parental education was included as a covariate in their analyses [52, 78]. However, the corresponding data were not presented alongside the study outcomes. Three studies (5%) provided vague categorization of parental education level (e.g., high school or below) [69, 88, 102]. Seven studies (11%) provided the specific parental education levels. Across these studies, 26 parents received no formal education or only primary schooling, 1,599 completed middle or high school, and 1,725 attained a university-level education.

### Household income

Household income was reported in ten studies (15%). One study (2%) stated that all participants came from families with similar household income levels without providing data [57]. One study (2%) used housing prices as a proxy of family financial status [93], and two studies (3%) reported mean household income [79, 97], all of which were insufficient to classify participants into income categories. Six studies categorised household income into low, medium, and high levels, with 2562, 2516, and 1714 participants in each category, respectively.

### Plus

Twenty-four studies (37%) included children living with health issues (*n* = 41, 63%). Seven studies focused on children living with overweight and/or obesity, nine involved participants with mental health issues, and eight included individuals with diseases or disabilities.

## Discussion

The present scoping review summarises the evidence on equity measures included in studies evaluating PA interventions among children and adolescents conducted in China. Our findings reveal critical issues in such studies that may exacerbate inequities. These include a highly disproportionate distribution of PA interventions across Chinese cities and provinces, and a greater likelihood of males being included compared with females. Children from less advantaged family background (e.g., lower parental education level) were underrepresented. Ethnic minoritised groups (e.g., Uyghur, Zhuang and Hui), as well as children who are displaced or left behind, were largely overlooked and may experience discrimination, based upon the limited available evidence. While many studies (*n* = 24, 37%) focused their attention on children with health issues, they did not directly aim to address PA inequities.

Our findings suggest that existing interventions may have contributed to widening PA inequities between children and adolescents living in rural versus urban areas, as well as across different city tiers. Only two interventions were conducted in rural areas, despite the fact that more than 36% of Chinese population residing in rural regions according to the 2021 population census [103]. In addition, most interventions were conducted in well-developed coastal cities, such as Beijing, Shanghai and Guangzhou. This disproportionate distribution is likely attributable to the use of convenience sampling, as 52 out of 65 interventions were carried out in locations affiliated with the leading researchers. Children living in well-developed cities benefit from greater PA-related resources, including greater availability of recreational infrastructure (e.g., bike lanes, parks, swimming pools), better air quality, and more human resources [27, 104, 105]. Indeed, compared to their rural counterparts in China, children in urban regions showed higher levels of PA and lower sedentary time [19, 20], as well as better muscular strength, cardiorespiratory fitness, and lower rates of overweight and obesity [106]. Therefore, greater attention, effort, and financial and political support are needed for not only the disadvantaged and underrepresented children in rural areas, but also for those working to support these communities.

There is also a notable lack of PA interventions focusing on ethnic minoritised groups and displaced children. China is a nation with 55 officially recognised ethnic minoritised groups, representing approximately 10% of the population [107]. These minority groups typically reside in remote areas and less developed provinces, and represent a wide range of socioeconomic backgrounds, languages, religions, and cultural and geographical contexts [108]. It is reported that children and adolescents of ethnic minority had higher mortality rates and lower immunisation coverage than Han children [107]. Despite these vulnerabilities, participant ethnicity was largely overlooked in the reviewed PA interventions, with only one intervention specifically targeting an ethnic minority population – the Uyghur group [33].

Even within Han population, there are culture and language differences across regions. A large number of Chinese adults temporarily moved from rural areas or less developed cities to large coastal cities for work, typically returning home only during major holidays such as the Chinese New Year. This internal migration has resulted in an estimated 90 million children who were left-behind or displaced [103]. Children who were left-behind remain in their home communities – typically cared for by grandparents, while in contrast, displaced children move with their parents to urban areas. Although their circumstances differ, both groups are vulnerable to psychological stress, bullying, and social exclusion, with left-behind children due to prolonged parental absence, and displaced children due to migration-related stigma and adjustment difficulties. Consequently, these children show higher risks of mental health problems and adverse emotional, social, and academic outcomes [109, 110]. Nevertheless, only one intervention has conducted among children who were left behind [101]. Surprisingly, one intervention even excluded children who could not speak the local language [53]. Although the rationale for this exclusion was not provided, such criteria may have inadvertently exacerbated PA inequities between migrant and local children.

Females are consistently reported to engage in less PA as compared to males, contributing to sex-based inequities in PA participation [19]. In the current review, male participants slightly outnumbered females (22398 vs. 20832), and the number of male-only interventions exceeded those exclusively targeting females (3 vs. 1). This disproportionate representation in PA interventions may further exacerbate existing sex disparities. Given that males often exhibit higher motivation for PA [111], future interventions should place greater emphasis on engaging female participants and addressing the specific barriers they face in accessing and participating in PA programs. A substantial number of interventions (25 out of 65) targeted populations subject to discrimination or stigma – such as children and adolescents who are overweight/obese or living with mental health conditions. This may reflect the rising prevalence of these issues among Chinese youth in recent years [112]. Although none of the interventions aimed to address PA inequity, focusing on vulnerable populations may indirectly contribute to reducing disparities in PA access and participation between children and adolescents with health issues and their peers [14].

Occupation, education, and household income are closely interrelated components of socioeconomic status, which can strongly influence PA participation. Lower socioeconomic status is consistently associated with reduced PA levels, as well as poorer psychological and physical health outcomes [113, 114]. However, only a small proportion of the reviewed interventions reported socioeconomic status related data. The available evidence revealed that fewer participants were recruited from families with unemployed parents, service workers, or lowest levels of parental educational attainment. This indicates that children from the most disadvantaged social backgrounds were less likely to be included in PA interventions, highlighting a critical equity gap in PA programme reach.

### Pathways toward equitable physical activity interventions

Children and adolescents deserve equal opportunities to engage in PA, thereby promoting health equity regardless of their place of residence, ethnicity, sex, health status, or family background. Despite claims for eliminating health inequity [24–26], the current review found no PA interventions explicitly designed to reduce PA disparities across the young population groups in China. This is consistent with findings from other countries reporting a general lack of equity considerations in PA interventions [115, 116]. To advance equity, future interventions must be designed and evaluated through an explicit equity and implementation lens.

Drawing on the RE-AIM framework [16], equity-oriented interventions should not only achieve reach across diverse groups but also demonstrate adoption, implementation, and maintenance in real-world settings. Well-controlled RCTs show limited potential to enhance equity if they are not adopted by schools or communities, implemented with fidelity, or sustained beyond the study period. Without intentional strategies to support these dimensions, even effective interventions may have little population-level impact.

Several strategies should therefore be considered to promote equity in future PA interventions. First and foremost, PA interventions should be designed with scale-up in mind [117]. Large-scale approaches can enhance reach by engaging diverse groups of children across marginalised and underserved populations [118]. Additionally, when resources are limited, interventions should prioritise children who are at-risk, underrepresented or disadvantaged, including those living with health issues, from rural and remote areas, from ethnic minoritised groups and from low socioeconomic backgrounds, or who are left behind or displaced. Furthermore, offering the exercise sessions to the control group upon completion of the intervention phase – an approach used in some of the included studies [59, 65, 81] – may help mitigate inequitable access to PA opportunities. Lastly, it is reported that “upstream” interventions focusing on social or policy level determinants, such as the one-hour PA policy in China, may work better at reducing PA inequities as compared to “downstream” interventions [12].

### Strengths and limitations

The present review is the first to evaluate inequities in accessing PA interventions in Chinese children and adolescents. A comprehensive assessment of equity measures was conducted using the PROGRESS-Plus framework. Several limitations within this scoping review should be acknowledged. First, we excluded publications written in Chinese, which is a major limitation given a substantial number of intervention studies are published in Chinese journals. This exclusion may have led to an underestimation of both the number and diversity of PA interventions implemented across the country. Although similar trends are expected, this assumption needs to be confirmed through further investigation. Second, the extent to which these interventions may have influenced health inequities remains unknown, as the effectiveness of interventions was not discussed in this review. Thirdly, the current review only examined the intervention reach of different population groups, while PA inequity may arise through other pathways, such as intervention uptake and compliance. Last but not the least, there was limited reporting across many equity measures, which constrains the strength of our conclusions.

## Conclusions

Equity considerations are largely absent from PA interventions involving children and adolescents conducted in China. As a result, these interventions may have unintentionally widened disparities in PA participation and, if effective, could contribute to broader health inequities. Future efforts should apply an equity lens in PA interventions by prioritizing underserved children and considering upstream, policy-level strategies to promote fair and inclusive PA opportunities.

## Supplementary Information

Supplementary Material 1.

## Data Availability

All data generated or analysed during this study are included in this published article.

## Abbreviations

PA: Physical activity
NG: Not given
RCT: Randomised controlled trial
SD: Standard deviation
PE: Physical education
QoL: Quality of life
FMD: Fundamental movement skill
ADHD: Attention deficit hyperactivity disorder
VR: Virtual reality
