# Supplementary material for: Physical activity interventions among children and adolescents in China: a scoping review through an equity lens
Source: Int J Behav Nutr Phys Act. 2025 Dec 22;23:9. doi: 10.1186/s12966-025-01866-w (PMC12838491; doi:10.1186/s12966-025-01866-w)
Supplement: Supplementary file 1 — Supplementary Material 1. [file 12966_2025_1866_MOESM1_ESM.docx]

**SPORTDiscus (EBSCOhost)**

S1 SU physical activity

S2 TI “physical activity” or train* or exerci* or sport*

S3 AB “physical activity” or train* or exerci* or sport*

S4 S1 OR S2 OR S3

S5 TI child* or adolescen* or youth# or teen* or student# or boy# or girl#

S6 AB child* or adolescen* or youth# or teen* or student# or boy# or girl#

S7 S5 OR S6

S8 TI China or Chinese

S9 AB China or Chinese

S10 S8 OR S9

S11 S4 AND S7 AND S10

S12 further limited to academic journals and English language

**Web of science**

1 TS=(physical activity)

2 TI=(“physical activity” or train* or exerci* or sport*)

3 AB=(“physical activity” or train* or exerci* or sport*)

4 #1 OR #2 OR #3

5 TI=(child* or adolescen* or youth$ or teen* or student$ or boy$ or girl$ )

6 AB=(child* or adolescen* or youth$ or teen* or student$ or boy$ or girl$)

7 #5 OR #6

8 TI=(China or Chinese)

9 AB=( China or Chinese )

10 #8 OR #9

11 #4 AND #7 AND #10

Document type: articles, languages: English

**SCOPUS**

( TITLE-ABS ( "physical activity" ) OR TITLE-ABS ( train* ) OR TITLE-ABS ( exerci* ) OR TITLE-ABS ( sport* ) ) AND ( TITLE-ABS ( child* OR adolescen* OR youth# OR teen* OR student# OR boy# OR girl# ) ) AND ( TITLE-ABS ( China ) OR TITLE-ABS ( Chinese ) ) AND ( LIMIT-TO ( DOCTYPE , "ar" ) ) AND ( LIMIT-TO ( LANGUAGE , "English" ) )

**Medline (Ovid)**

1 exp Physical activity/

2 (“physical activity” or train* or exerci* or sport*).tw,kw.

3 Or/1-2

4 exp Child/

5 Child*.mp.

6 exp Adolescent/

7 Adolescen*.mp.

8 (youth? or teen* or boy? or girl?).mp.

9 student?.mp.

10 or/4-9

11 (China or Chinese). tw,kw.

12 and/3,10,11

13 limit 12 to (english language and humans)

**Cochrane Central Register of Controlled Trials**

#1 [mh ^"physical activity"]

#2 (“physical activity” or train* or exerci* or sport*):ti,ab,kw

#3 #1or #2

#4 (child* or adolescen* or youth? or teen* or student? or boy? or girl? ):ti,ab,kw

#5 (China or Chinese) :ti,ab,kw

#6 {AND #3-#5}
